# Characterization of the complete chloroplast genome of *Pteris multifida* Poir. 1804 and its phylogenetic analysis

**DOI:** 10.1080/23802359.2021.2002209

**Published:** 2021-11-24

**Authors:** Yufei Wang, Yuan Jiang, Jing Wang, Xiaoli Yang, Jun Qian

**Affiliations:** College of Pharmaceutical Science, Dali University, Dali, China

**Keywords:** *Pteris multifida* Poir. 1804, complete chloroplast genome, phylogenetic analysis

## Abstract

*Pteris multifida* Poir. 1804 has a long history of medicinal use in China. The chloroplast (cp) genome of *P*. *multifida* was 153,916 bp in length, with a large single-copy (LSC) region of 82,027 bp, a small single-copy (SSC) region of 21,129 bp, and a pair of inverted repeats (IRs) of 25,380 bp, forming a typical quadripartite structure. A total of 132 genes were annotated from the cp genome of *P*. *multifida*, including 89 protein-coding genes, 35 tRNA genes, and eight ribosomal RNA genes. Phylogenetic analysis indicated that *P*. *multifida* was closely related to the species of *P*. *vittata.*

*Pteris multifida* Poir. 1804 (Huang 1997), a herbaceous plant that belongs to the *Pteris* genus in the Pteridaceae family, is distributed widely near wells, wall edges, or under bushes (Zhang et al. 2021). The whole plant has been used to treat jaundice, dysentery, hematochezia, eczema, and other diseases in Chinese medicine (Chen et al. 2018). Previous studies mainly focused on pharmacological activity, chemical composition (Yu et al. 2001; Hu and Zheng 2005). However, there is no genomic report on *P*. *multifida* to date. Herein, we assembled and characterized the complete chloroplast (cp) genome of *P. multifida*, which will provide a valuable framework for phylogenetic and evolutionary studies in Pteridaceae.

The fresh leaves were collected from Yangbi county, Yunnan Province (25°40′11.71″,N, 99°57′29.30″E), and the voucher specimen was deposited in the herbarium of Dali University (herbarium code: Y706, contact person: Baozhong Duan, email: bzduan@126.com) under the voucher number HBGP0422. The total DNA was isolated using the DNeasy plant mini kit (QIAGEN). The Paired-end library was sequenced using the Illumina NovaSeq platform (San Diego, CA). Approximately 5.95 Gb of raw data (20,309,333 reads) was assembled by NOVOPlasty v3.7 (Dierckxsens et al. 2017) with complete cp genome of *Pteris vittata* as the reference (GenBank accession No. MH173082). Gene annotation was conducted using GeSeq based on default parameters and manually checked for errors. (Tillich et al. 2017; Yang et al. 2020). Finally, the annotated cp genome has been submitted to the GenBank at the National Center of Biotechnology Information (NCBI) under specific accession numbers of MZ848380.

The cp genome sequence of *P*. *multifida* was 153,916 bp in length and exhibited a typical quadripartite structure, including a large single copy (LSC) region of 82,027 bp, a small single copy (SSC) region of 21,129 bp, and a pair of inverted repeat (IRs) regions of 25,380 bp. The overall GC content of the cp genome is 42.2%. A total of 132 genes were annotated, including 89 protein-coding regions located in the LSC, 35 coding regions in the IR, and eight regions in the SSC.

To identify the phylogenetic position of *P*. multifida, 20 species of Pteridaceae family from NCBI were aligned using MAFFT v7.309 (Katoh and Standley 2013). *Goniophlebium amoenum* and *G*. *niponicum* were served as the out-group. The phylogenetic analysis was conducted based on maximum likelihood (ML) with IQtree (Nguyen et al. 2015), bootstrap probability values were calculated from 1000 replicates. The phylogenetic tree showed that *P*. *multifida* was closely related to *P*. *vittata* with strong bootstrap support. This result was consistent with the findings of PPG I (Figure 1) (PPGI 2016). Thus, adding cp genomic data of this species within section Pteridaceae would provide further insights into chloroplast evolution and potential gene flow among species in the group.

**Figure 1.**
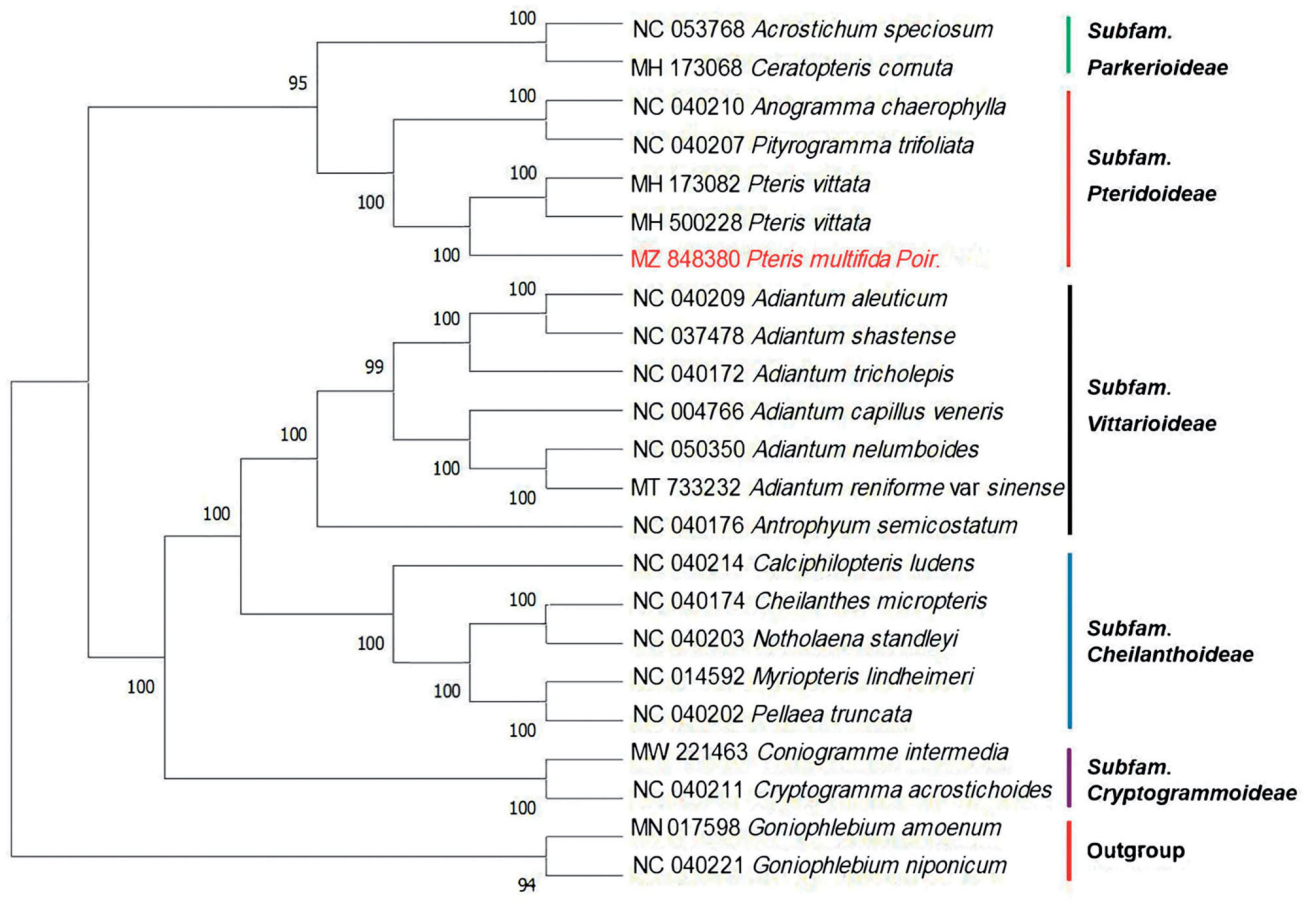
The Maximum-likelihood phylogenetic tree based on the cp genome of 23 species. The bootstrap values were 1000 replicates.

## Data Availability

The data supporting this study were openly available in NCBI, under GenBank accession number MZ848380. The NGS sequencing data files were available from the BioProject, SPR, and Bio-Sample ID under the accession numbers PRJNA753886, SRR15422630, and SAMN20717418, respectively.
